# Single-cell spatiotemporal analysis of the lungs reveals *Slamf9*^+^ macrophages involved in viral clearance and inflammation resolution

**DOI:** 10.1038/s41421-024-00734-4

**Published:** 2024-10-16

**Authors:** Boyi Cong, Xuan Dong, Zongheng Yang, Pin Yu, Yangyang Chai, Jiaqi Liu, Meihan Zhang, Yupeng Zang, Jingmin Kang, Yu Feng, Yi Liu, Weimin Feng, Dehe Wang, Wei Deng, Fengdi Li, Zhiqi Song, Ziqiao Wang, Xiaosu Chen, Hua Qin, Qinyi Yu, Zhiqing Li, Shuxun Liu, Xun Xu, Nanshan Zhong, Xianwen Ren, Chuan Qin, Longqi Liu, Jian Wang, Xuetao Cao

**Affiliations:** 1 State Key Laboratory of Medicinal Chemical Biology, Institute of Immunology, College of Life Sciences, Nankai University, Tianjin, China; 2 Department of Immunology, Center for Immunotherapy, Peking Union Medical College, Chinese Academy of Medical Sciences, Beijing, China; 3 BGI-Shenzhen, Shenzhen, Guangdong China; 4 Institute of Laboratory Animal Sciences, Chinese Academy of Medical Sciences, Beijing, China; 5 Changping Laboratory, Beijing, China; 6 Institute of Immunology, Zhejiang University School of Medicine, Hangzhou, Zhejiang China; 7 National Key Laboratory of Immunity and Inflammation, Institute of Immunology, Navy Medical University, Shanghai, China; 8 Guangzhou Laboratory, Guangzhou, Guangdong China

**Keywords:** Innate immunity, Bioinformatics

## Abstract

How the lung achieves immune homeostasis after a pulmonary infection is not fully understood. Here, we analyzed the spatiotemporal changes in the lungs over a 2-week natural recovery from severe pneumonia in a Syrian hamster model of SARS-CoV-2 infection. We find that SARS-CoV-2 infects multiple cell types and causes massive cell death at the early stage, including alveolar macrophages. We identify a group of monocyte-derived *Slamf9*^+^ macrophages, which are induced after SARS-CoV-2 infection and resistant to impairment caused by SARS-CoV-2. *Slamf9*^+^ macrophages contain SARS-CoV-2, recruit and interact with *Isg12*^+^*Cst7*^+^ neutrophils to clear the viruses. After viral clearance, *Slamf9*^+^ macrophages differentiate into *Trem2*^+^ and *Fbp1*^+^ macrophages, contributing to inflammation resolution at the late stage, and finally replenish alveolar macrophages. These findings are validated in a SARS-CoV-2-infected hACE2 mouse model and confirmed with publicly available human autopsy single-cell RNA-seq data, demonstrating the potential role of *Slamf9*^+^ macrophages and their coordination with neutrophils in post-injury tissue repair and inflammation resolution.

## Introduction

Lung injury is commonly accompanied with viral or bacterial infection. Studies have reported the physiological roles of alveolar epithelial cells and macrophages in the post-injury lung tissue regeneration^1,2^. However, it is still unknown how immune homeostasis is maintained spatiotemporally in lung. The continuously emerging and evolving SARS-CoV-2 variants have caused multiple waves of pulmonary infections worldwide^3^. Multiple studies have analyzed the innate and adaptive immune profiles based on multiple tissues and blood samples of COVID-19 patients, shedding light on the immunological and immunopathological mechanisms for inflammation initiation and tissue damage^4–10^. SARS-CoV-2 viral RNA has been detected in multiple types of immune cells, including both myeloid cells (neutrophils, monocytes, and macrophages) and lymphocytes (T, B, and natural killer (NK) cells), to initiate wide immune responses and cytokine storms^5,9^. Antibody-mediated uptake of SARS-CoV-2 by monocytes and macrophages can trigger inflammatory cell death, thereby leading to systemic inflammation that contributes to the pathogenesis of COVID-19^11^. However, inflammation resolution and immune homeostasis remain unaddressed, as these human studies were limited by the types of samples available, including autopsies, peripheral blood, and bronchoalveolar lavage fluid. Although experimental animals including human ACE2 transgenic mice (hACE2 mice) and hamsters have been used to model the pathogenesis of SARS-CoV-2 infection^12–15^, the absence of high-resolution spatiotemporal information of the molecular and cellular changes in SARS-CoV-2 pulmonary infection made most of the current studies segmentary, and thus the mechanisms from SARS-CoV-2 pathogenesis, immune escape, viral clearance, to inflammation resolution and immune homeostasis are still elusive.

Identifying cell subpopulations responsible for clearing SARS-CoV-2, resolving pulmonary inflammation, and maintaining immune homeostasis is pivotal to understand COVID-19 pathogenesis and developing antiviral therapeutics against lung injury^16^. Here we established a model to simulate severe pneumonia and the viral clearance and inflammation resolution process by infecting Syrian hamsters with appropriate titers of authentic SARS-CoV-2. Syrian hamsters, naturally susceptible to SARS-CoV-2 infection, have been widely used in hundreds of studies to mimic moderate or severe COVID-19 with the aim of understanding the pathogenesis of SARS-CoV-2 infection, developing and evaluating the effectiveness of new drugs, vaccines, and antibodies, providing rich resources for further investigation^13,17–23^. We applied Stereo-seq^24^ for spatial transcriptomics together with droplet-based high-throughput single-cell RNA sequencing (scRNA-seq) to the lungs of 25 hamsters infected with or without authentic SARS-CoV-2. We generated a comprehensive spatiotemporal cellular and molecular atlas of the whole lung lobes spanning the entire course of SARS-CoV-2 infection, ranging from tissue damage to severe pneumonia, and to viral clearance and inflammation resolution, enabling discoveries of novel mechanisms related to host responses and immune homeostasis. We identified that a group of *Slamf9*^+^ macrophages in the lung, which were specifically induced from monocytes during the acute infection phase and kept active proliferation, could recruit neutrophils to collaboratively clear SARS-CoV-2 from hamster lungs. After viral clearance, *Slamf9*^+^ macrophages differentiated into *Trem2*^+^ and *Fbp1*^+^ macrophages to resolve inflammation, replenish alveolar macrophages, and repair damaged lungs. The data resource of the spatial dynamics of pulmonary infection and the identification of *Slamf9*^+^ macrophages for viral clearance and inflammation resolution provide valuable information for controlling pulmonary infection.

## Results

### Single-cell landscape of lung cell subpopulations during SARS-CoV-2 infection

**Fig. 1:**
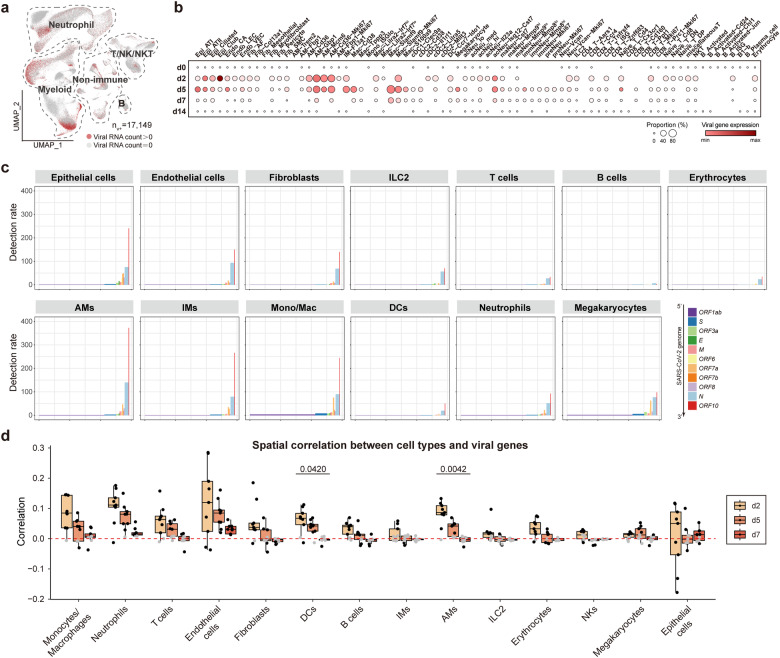
Signatures of multiple cell types after SARS-CoV-2 infection. **a** UMAP projection displaying the distribution of 17,149 SARS-CoV-2 RNA-positive cells (n_v+_, number of total virus-positive cells detected). **b** Dot plot showing the relative proportion of virus-positive cells and the expression of viral genes within 79 cell subpopulations. Data are from two replicated hamsters. **c** Detection rates of SARS-CoV-2 genes in main cell types. Given a viral gene *g*_*v*_, the detection rate is defined as the ratio of the number of *g*_*v*_^+^ cells to the total cells of the specific cell type and then normalized by the gene length in the SARS-CoV-2 genome and multiplied by 10^6^. **d** Box plot showing the spatial correlation between different cell types and viral genes of nine lung slides at d2, d5, and d7. Data are represented as mean ± SEM (*n* = 9 slides per timepoint). Center line, median; box bounds, first and third quartiles; whiskers, 1.5 times the interquartile range. Kruskal–Wallis test was performed to calculate the *P* value across different timepoints. Pearson correlation was performed to calculate the *P* value of all spots within each slide. Black dots: *P* < 0.05; gray dots: *P* ≥ 0.05. Red dotted line represents the correlation value of 0.

To map the whole process of SARS-CoV-2 pulmonary infection and clearance, we obtained a comprehensive cellular and molecular atlas using Stereo-seq combined with scRNA-seq (the technical details can be found in our companion paper on dendritic cell–T immunity hubs^25^). These cell subpopulations together with the detection of SARS-CoV-2 RNAs demonstrated different dynamics during the infection process (Fig. 1a, b; Supplementary Fig. S1a, b). Dramatic temporal changes were observed for SARS-CoV-2 RNA-positive cells (Fig. 1b; Supplementary Fig. S1a, b), consistent with previous observations in hamsters^13^. Multiple cell types, including non-immune cells and specific clusters of macrophages, dendritic cells (DCs), T cells, and B cells, demonstrated a decreasing trend from d2 to d7 and then partially restored at d14, which were consistent with the observations of tissue damages and the following alleviation (Supplementary Fig. S1a). The lung-resident alveolar macrophages (AMs) are critical innate pulmonary sentinels for respiratory pathogens, playing important roles in homeostasis maintenance and pathogen clearance. Similarly, lung-resident AMs started to decrease after d2, and partially restore at d14 (Supplementary Fig. S1a), indicating that SARS-CoV-2 infection damaged the lung-resident AMs to escape lung innate immune defense. In contrast, most clusters of monocytes/macrophages and neutrophils were enriched at d5 and d7, exhibited huge phenotypic heterogeneity, and demonstrated profound temporal changes (Supplementary Fig. S1a).

As expected, viral RNAs were detected in a high proportion of epithelial cells (Fig. 1b). In addition to epithelial cells previously regarded as the main host cells of Sarbecoviruses^26,27^, we found that diverse pulmonary cell types were also SARS-CoV-2 RNA-positive (Fig. 1a, b; Supplementary Fig. S1b), consistent with previous observations based on human autopsy, bronchoalveolar lavage fluid and sputum samples as well as hamster lungs^5,9,14,28,29^. Coronaviruses are characterized by subgenomic transcription, during which subgenomic RNAs, i.e., RNAs sharing the same 3’ ends as the genomic RNA but with 5’ ends near the start codons of downstream open reading frames (ORFs), are transcribed instead of ORFs^30^. Active subgenomic transcription of SARS-CoV-2 was indicated in multiple cell types in our data, as evidenced by the detection rate of SARS-CoV-2 genome increasing from 5’ to 3’ (Fig. 1c). Despite such a global trend, the detection rates of SARS-CoV-2 RNA varied among cell subpopulations. AMs, interstitial macrophages (IMs), monocytes/macrophages, neutrophils, and non-immune cells including endothelial cells and fibroblasts were also characterized by the high subgenomic transcription of SARS-CoV-2 (Fig. 1c), with the detection rates similar to or even exceeding those in epithelial cells. DCs, T, and B cells harbored a relatively lower detection rate, particularly in plasma cells (Fig. 1c).

### SARS-CoV-2 infection of lung-resident macrophage subpopulations and the accompanying cell death

**Fig. 2:**
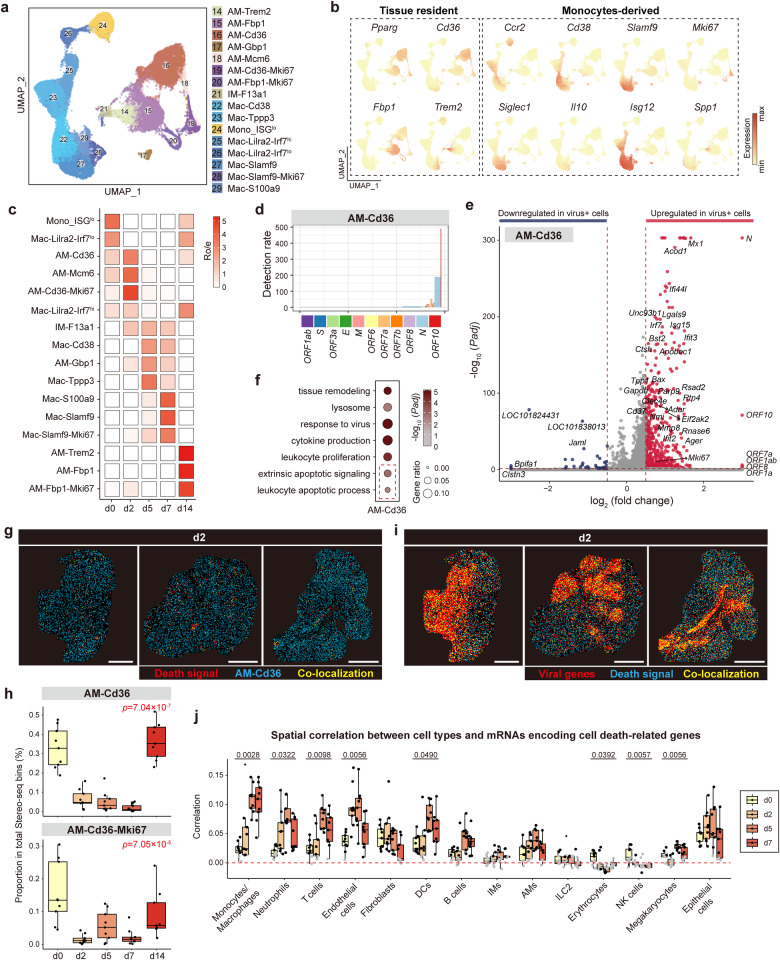
SARS-CoV-2 infection causes massive death of lung-resident cells at the early stage. **a** UMAP projection of AM and monocyte/macrophage showing 15 cell subpopulations. **b** The expression of selected genes defining key tissue-resident AM and monocyte/macrophage subpopulations. **c** The temporal distribution of AM and monocyte/macrophage subpopulations. Ro/e > 1, enrichment; Ro/e < 1, depletion. **d** Detection rates of SARS-CoV-2 genes in AM-Cd36. Given a viral gene *g*_*v*_, the detection rate is defined as the ratio of the number of *g*_*v*_^+^ cells to the total cells of the specific cell type and then normalized by the gene length in the SARS-CoV-2 genome and multiplied by 10^6^. **e** Volcano plot of differentially expressed genes between virus-positive and virus-negative cells in AM-Cd36 (cutoff: (|Log_2_FC| > 0.5, *Padj* < 0.05)). **f** Gene ontology (GO) terms enriched in upregulated genes in virus-positive AM-Cd36. GO terms related with cell death are indicated by dashed line. **g** Spatial distribution of cell death-related genes (death signal) and AMs, represented by three lung sections at d2. Scale bars, 2 mm. **h** Boxplot showing the proportion of AM-Cd36 and proliferating AM-Cd36 in total Stereo-seq bin80-bins of nine lung slides at each timepoint. Center line, median; box bounds, first and third quartiles; whiskers, 1.5 times the interquartile range. Data are represented as mean ± SEM (*n* = 9 slides per timepoint). Kruskal–Wallis test. **i** Spatial distribution of viral genes and death signals, represented by three lung sections at d2. Scale bars, 2 mm. **j** Boxplot showing the spatial correlation of different cell types and death-related genes at d0, d2, d5, and d7. Data are represented as mean ± SEM (*n* = 9 slides per timepoint). Center line, median; box bounds, first and third quartiles; whiskers, 1.5 times the interquartile range. Kruskal–Wallis test was performed to calculate the *P* value across different timepoints. Pearson correlation was performed to calculate the *P* value of all spots within each slide. Black dots: *P* < 0.05; gray dots: *P* ≥ 0.05. Red dotted line represents the correlation value of 0.

We calculated the spatial correlation between different cell types and viral genes based on our spatial transcriptomic data and found that endothelial cells, neutrophils, monocytes/macrophages, and AMs were most co-localized with viral genes at especially d2 (Fig. 1d). Among the 16 subpopulations of macrophages identified in this study, which demonstrated distinct temporal enrichment characteristics, a subpopulation of *Marco*^*+*^*Mrc1*^*+*^*Pparg*^*+*^*Ccr2*^*–*^ lung-resident AMs (AM-Cd36, cluster 16) was enriched in d0 and d2 (Fig. 2a-c), which possessed the highest proportion of virus-positive cells at d2 according to scRNA-seq data (up to 50%, much higher than 26% in epithelial cells; Supplementary Fig. S1b). This subpopulation was also characterized by active subgenomic transcription of SARS-CoV-2 implied by the increasing detection rates of SARS-CoV-2 genes along the genome from 5’ to 3’ (Fig. 2d), indicating active SARS-CoV-2 transcription. The infection status of AM-Cd36 was further supported by the transcriptional alterations observed between virus-positive and virus-negative cells in AM-Cd36, of which genes related to “response to virus” (such as *Mx1* and *Isg15)* and “apoptosis” (such as *Bax* and *Foxo3*) were upregulated (Fig. 2e, f). The in situ nature of Stereo-seq may capture more cell death-related events and thus is more suitable to investigate SARS-CoV-2-induced damages. AM-Cd36 cells were almost depleted from hamster lungs 2 days after SARS-CoV-2 infection, consistent with the enrichment of cell death-related genes within the neighborhood of SARS-CoV-2-positive AM-Cd36 macrophages (Fig. 2g, h). Viral genes were significantly correlated with death signals according to Stereo-seq data (Fig. 2i). In fact, it was reported that FcγR-mediated SARS-CoV-2 infection of human macrophages and monocytes can cause cell death and inflammation^11^, consistent with our observations based on single-cell and spatial transcriptomics.

Besides cell death-related genes, *Mki67* was also upregulated in virus-positive AM-Cd36 cells (Fig. 2e), and consequently, we identified a proliferating AM-Cd36 subpopulation termed as AM-Cd36-Mki67 (cluster 19) at d2, the early stage of infection (Fig. 2a–c). Spatially, the expression of *Mki67* was dispersed across the centimeter-scale lung sections, with SARS-CoV-2-dense and -sparse regions showing similar occurrence (Supplementary Fig. S2a), suggesting that the AM-Cd36 proliferation may be in situ triggered by the viruses as a quick response. Because AMs are the first line of pulmonary defense that survey the lumen of respiratory tracts, our observations suggested that in situ proliferation of AMs may be an important strategy for the local host innate immune system to defend SARS-CoV-2 invasion, which may form a quick response before the recruitment of potentially tissue-damaging inflammatory cells from distant sites^31^. However, massive viral particles may break the defense line imposed by AMs via inducing cell death of AMs (Fig. 2e–g).

In addition to type II alveolar epithelial cells (ATII), erythrocytes, club cells and AM-Cd36 macrophages, lymphatic endothelial cells (LECs), vein endothelial cells (VECs), fibroblast, neutrophils, IMs and natural killer T (NKT) cells were also responsible for SARS-CoV-2-induced acute tissue damages according to Stereo-seq and scRNA-seq data (Supplementary Fig. S2b–d). SARS-CoV-2-positive cells of these cell types upregulated genes related to cell death (Supplementary Fig. S2b–d), and multiple cell types were spatially correlated with cell death signals after viral infection according to Stereo-seq data (Fig. 2j). These data suggested that SARS-CoV-2 infection led to massive cell death across multiple cell types, including lung-resident AMs.

### The spatiotemporal characteristics of *Slamf9*^+^ macrophages in defense to SARS-CoV-2 infection

**Fig. 3:**
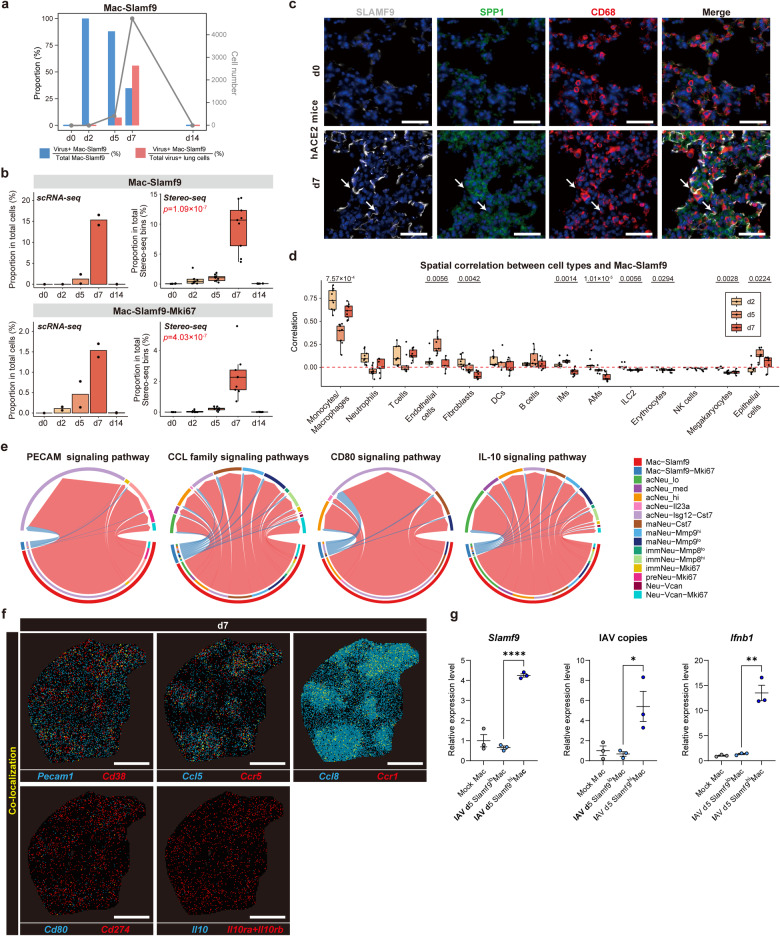
SARS-CoV-2-induced generation of *Slamf9*^+^ macrophages for viral clearance. **a** The number of total *Slamf9*^+^ macrophages, the proportion of virus-positive *Slamf9*^+^ macrophages, and their proportion in total virus-positive cells at each timepoint. **b** Left, bar chart showing the proportion of *Slamf9*^+^ macrophages and proliferating *Slamf9*^+^ macrophages in total lung cells detected in two replicated hamsters by scRNA-seq. Right, boxplot showing the proportion of *Slamf9*^+^ macrophages and proliferating *Slamf9*^+^ macrophages in total Stereo-seq bin80-bins of nine lung slides at each timepoint. Data are represented as mean ± SEM (*n* = 9 slides per timepoint). Center line, median; box bounds, first and third quartiles; whiskers, 1.5 times the interquartile range. Kruskal–Wallis test. **c** Representative images of the lung sections of hACE2 mice before and after SARS-CoV-2 infection detected by immunofluorescent staining. SLAMF9, SPP1 and CD68 at d0 and d7. SLAMF9^+^SPP1^+^ macrophages were indicated by arrows. Scale bars, 50 μm. **d** Boxplot showing the spatial correlation between different cell types and *Slamf9*^+^ macrophages of nine lung slides at d2, d5 and d7. Data are represented as mean ± SEM (*n* = 9 slides per timepoint). Center line, median; box bounds, first and third quartiles; whiskers, 1.5 times the interquartile range. Kruskal–Wallis test was performed to calculate the *P* value across different timepoints. Pearson correlation was performed to calculate the *P* value of all spots within each slide. Black dots: *P* < 0.05; gray dots: *P* ≥ 0.05. Red dotted line represents the correlation value of 0. **e** Representative interaction pathways among *Slamf9*^+^ macrophages, proliferating *Slamf9*^+^ macrophages and neurtophils subpopulations. The initiation of the chord represented the source subpopulations, and the end of the chord represented the targeted subpopulations. **f** Spatial expression of representative paired ligand–receptor genes, represented by a lung section at d7. Scale bars, 2 mm. **g** Relative mRNA expression level of *Slamf9*, IAV copies and *Ifnb1* detected in the SLAMF9^hi^ and SLAMF9^lo^ macrophages in the lung tissues of C57BL/6 J mice at 5 days after IAV infection. Unpaired two-sided Student’s *t*-tests (*n* = 3 indepe*n*dent experiments). *P < 0.05, **P < 0.01, ****P < 0.0001.

Accompanying the tissue damages caused by viral infection, two macrophage subpopulations with extremely high detection rates of SARS-CoV-2 genes accumulated in hamster lungs and became the major cell populations of SARS-CoV-2 RNA-positive cells (Supplementary Fig. S3a). At d2, *Cd38*^+^ macrophages (Mac-Cd38, cluster 22) and *Slamf9*^+^ macrophages (Mac-Slamf9, cluster 27) occupied a small proportion of myeloid cells within hamster lungs. In particular, only two cells were detected for *Slamf9*^+^ macrophages by scRNA-seq, but both were SARS-CoV-2 RNA-positive (Fig. 3a).

Notably, we identified a new macrophage subpopulation highly expressing *Slamf9, Spp1*, *Siglec1,* and *Il10*, also including a proliferating state (Figs. 2b, 3b). These *Slamf9*^+^ macrophages maintained an extremely high rate of SARS-CoV-2 RNA positivity at d5 (88%), and the cell number of this cluster increased drastically compared with that at d2, occupying 7.3% of all the SARS-CoV-2 RNA-positive cells at d5 (Fig. 3a). At d7, *Slamf9*^+^ macrophages further increased and became the major part of SARS-CoV-2 RNA-positive cells, but the SARS-CoV-2 RNA-positive rate of *Slamf9*^+^ macrophages decreased to 34.8%, occupying 56% of the total virus-positive cells (Fig. 3a). *Cd38*^+^ macrophages and *Slamf9*^+^ macrophages were both characterized by active subgenomic transcription of SARS-CoV-2 (Supplementary Fig. S3b). Accumulation of *Cd38*^+^ macrophages may indicate the severe pneumonia at d5 (Supplementary Fig. S3c), as CD38 has been reported to be an inflammatory marker of human monocytes and macrophages and involved in COVID-19 pathophysiology^32,33^. Functional enrichment analysis based on gene ontology revealed that virus-positive *Slamf9*^+^ macrophages primarily upregulated genes related to “tissue remodeling” and “vascular endothelial growth factor production”, but not genes associated with cell death-related genes compared to tissue-resident AMs (Supplementary Fig. S3d, e), implying cell death-resistant phenotypes of these macrophages and their potential roles in resolving inflammation and viral infection by pro-fibrosis. Noticing the disappearance of *Slamf9*^+^ and *Cd38*^+^ macrophages after SARS-CoV-2 clearance at d14 (Fig. 3b; Supplementary Fig. S3c), we speculated that the specific transcriptional state of *Slamf9*^+^ macrophages may be induced by engulfment or infection of SARS-CoV-2, which needs to be further investigated. The existence of *Slamf9*^+^ macrophages was further supported by immunofluorescent staining in the lung sections of hACE2-mice infected by SARS-CoV-2 (Fig. 3c). To further verify the presence of the proliferating *Slamf9*^+^ macrophages, we carried out in situ RNA hybridization, and observed the co-localization of *Cd68, Il10* and *Mki67* RNA (Supplementary Fig. S3f).

Endothelial cells and epithelial cells showed increased spatial co-localization with *Slamf9*^+^ macrophages from d2 to d5 (Fig. 3d), suggesting recruitment of macrophages to the injured sites. Noticeably, neutrophils spatially surrounding *Slamf9*^+^ macrophages increased from d5 to d7 (Fig. 3d). To provide unbiased confirmation of cross-connections between macrophages and neutrophils crosstalk, we performed computational intercellular interaction analyses via CellChat. Ligand–receptor analysis based on scRNA-seq data suggested that *Slamf9*^+^ macrophages may have distinct interactions with different neutrophil subpopulations (Supplementary Fig. S4a, b). Specifically, *Slamf9*^+^ macrophages might interact with different neutrophil subpopulations via PECAM pathway, CCL pathway, CD80 pathway, IL-10 pathway, etc. (Fig. 3e). Co-expression of the representative ligand–receptor genes, including *Pecam1/Cd38*, *Ccl5/Ccr5*, *Ccl8/Ccr1*, *Cd80/Cd274,* and *Il10/Il10ra+Il10rb* could also be detected according to our Stereo-seq data (Fig. 3f), suggesting the potential recruitment and functional regulation of neutrophils by *Slamf9*^+^ macrophages. The interaction via the inhibitory signaling pathways also implied the role of *Slamf9*^+^ macrophages in combating SARS-CoV-2 infection and leading to inflammation resolution together with neutrophils (Fig. 3e, f). We also proved in C57BL/6 mice that the viral load was significantly higher in SLAMF9^hi^ macrophages than SLAMF9^lo^ macrophages sorted from the lung tissues at 5 days after influenza virus (IAV) infection. SLAMF9^hi^ macrophages also expressed higher levels of interferon-β (*Ifnb1*). These results further suggest the potential function of SLAMF9^+^ macrophages to clear virus (Fig. 3g; Supplementary Fig. S4c). Besides, *Slamf9*^+^ macrophages were spatially co-localized with DC–T immunity hubs, which might coordinate to eliminate viral infection in the lung (the details can be found in our companion paper on DC–T immunity hubs^25^).

### Specific generation of *Isg12*^+^*Cst7*^+^ neutrophils in lung for clearing SARS-CoV-2

**Fig. 4:**
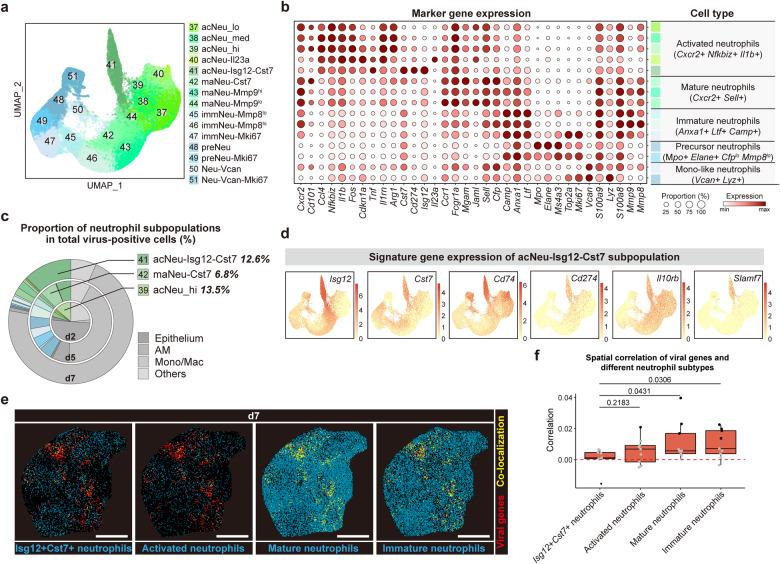
*Isg12*^*+*^*Cst7*^*+*^ neutrophils accumulate for eliminating viruses. **a** UMAP projection showing 15 neutrophil subpopulations. **b** Dot plot showing the expression of signature genes defining different neutrophil subpopulations. **c** The proportion of each cell subpopulation of virus-positive neutrophils in total virus-positive cells in d2, d5, and d7. **d** UMAP projection showing the expression of signature genes identifying acNeu-Isg12-Cst7 subpopulation. **e** Spatial distribution of viral genes and different neutrophil subtypes, represented by a lung section at d7. Scale bars, 2 mm. **f** Box plot showing the correlation of viral genes and different neutrophil subtypes. Data are represented as mean ± SEM (*n* = 9 slides per timepoint). Center line, median; box bounds, first and third quartiles; whiskers, 1.5 times the interquartile range. Statistical analysis between two groups was performed by two-sided Student’s *t*-test. Statistical analysis within each slide was performed by Pearson correlation, where black dots represented *P* < 0.05, and gray dots represented *P* ≥ 0.05.

During the acute phase of inflammation, neutrophils are generally believed to be recruited from peripheral blood to inflamed sites to clear infection or tissue injuries^34^. Consistently, we observed that the hamster lungs were replenished with peripherally derived neutrophils and demonstrated high phenotype diversity. A total of 15 subpopulations was identified for neutrophils based on our scRNA-seq data, including precursor, immature, mature, activated neutrophils, and two subpopulations with high levels of *Vcan* and *Lyz* (Fig. 4a, b). Similar to macrophage subsets, these neutrophil subpopulations with different cellular and functional states also demonstrated distinct temporal patterns (Supplementary Fig. S5a, b).

Similar to AM-Cd36, lowly-activated neutrophils (acNeu_lo) diminished after infection (Supplementary Fig. S5c). Highly-activated neutrophils (acNeu_hi) were the major population of SARS-CoV-2 RNA-positive neutrophils at d2 (Fig. 4c). SARS-CoV-2 RNA-positive highly-activated neutrophils upregulated genes related to IL-6 production, type I IFN production and cell death (Supplementary Fig. S5d), and the number of such acNeu_hi decreased at d5 and d7 (Supplementary Fig. S5c).

We noticed that a subpopulation of neutrophils characterized by high expression of *Isg12, Cst7, Cd74, Slamf7, and Cd274* (acNeu-Isg12-Cst7) were enriched at d7 and formed the major part of total virus-positive neutrophils (Fig. 4c, d). CST7 (cystatin F), a cysteine peptidase inhibitor, was reported to suppress cytotoxicity of T cells and NK cells^35^. ISG12 (interferon-alpha inducible protein 27) was reported to regulate innate inflammatory responses and also inhibit viral replication^36,37^. A negative correlation was observed between the proportion of virus-positive total cells and that of *Isg12*^+^*Cst7*^+^ neutrophils (Supplementary Fig. S5e), and virus-positive *Isg12*^+^*Cst7*^+^ neutrophils upregulated genes related to vesicle organization and vesicle-mediated transport (Supplementary Fig. S5d). Based on the spatial transcriptomic data, we found that, unlike other types of neutrophils located near the virus, *Isg12*^+^*Cst7*^+^ neutrophils were positioned farther away from viral genes (Fig. 4e, f; Supplementary Fig. S5f), implying the potential function of *Isg12*^+^*Cst7*^+^ neutrophils in eliminating the surrounding viruses or virus-infected cells. RNA velocity analysis suggested that *Isg12*^+^*Cst7*^+^ neutrophils may be derived from neutrophils recruited from peripheral blood instead of lung-resident neutrophils (Supplementary Fig. S5g). Moreover, *Isg12*^+^*Cst7*^+^ neutrophils expressed *Cd274* and *Il10rb* (Fig. 4d), potentially interacted with activated cytotoxic T cells and *Slamf9*^+^ macrophages, and jointly established the favoring niches for the resolution of inflammation at the late stage of infection (Fig. 3e, f).

### Enrichment of *Trem2*^+^ and *Fbp1*^+^ macrophages in lung at the late stage of viral infection for inflammation resolution and alveolar macrophage replenishment

**Fig. 5:**
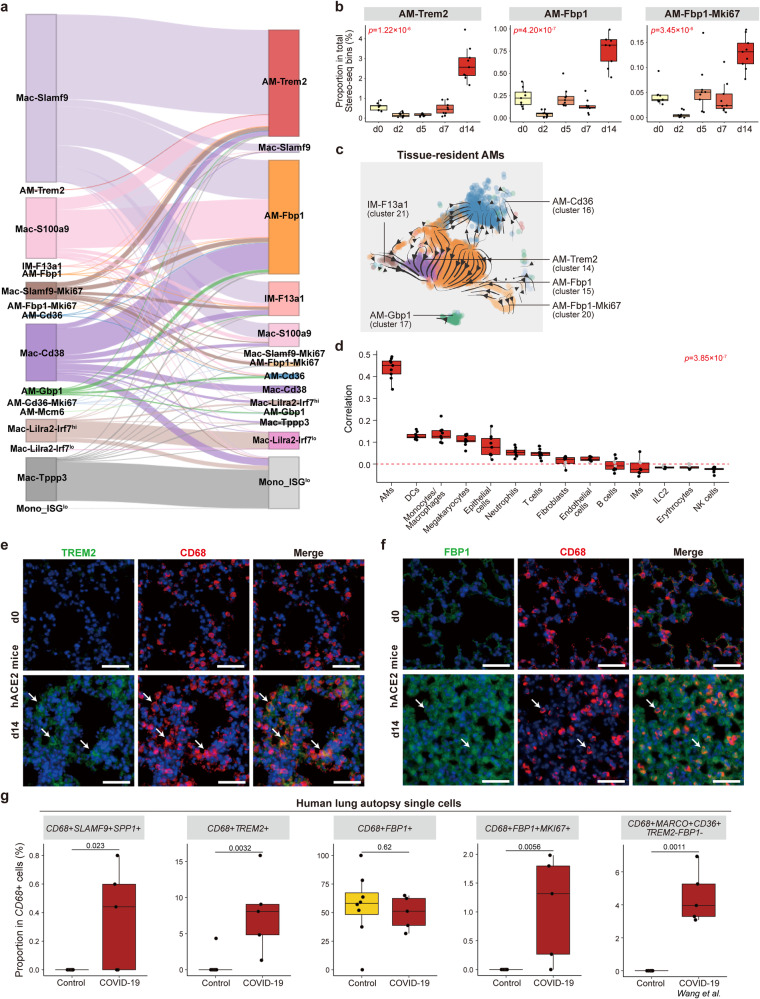
Enrichment of *Trem2*^+^ and *Fbp1*^+^ macrophages in the lung after SARS-CoV-2 clearance. **a** Cell fate inference analysis by CellRank based on monocyte/macrophage scRNA-seq data at d7 and d14. **b** Boxplot showing the proportion of *Trem2*^*+*^ AMs, *Fbp1*^+^ AMs and proliferating *Fbp1*^+^ AMs in total Stereo-seq bin80-bins of nine lung slides at each timepoint. Data are represented as mean ± SEM (*n* = 9 slides per timepoint). Center line, median; box bounds, first and third quartiles; whiskers, 1.5 times the interquartile range. Kruskal–Wallis test. **c** RNA velocity analysis of AM subpopulations. Arrows indicate the potential directions of state transitions. **d** Boxplot showing the spatial correlation between different cell types and *Fbp1*^+^ AMs of nine lung slides at d14. Data are represented as mean ± SEM (*n* = 9 slides per timepoint). Center line, median; box bounds, first and third quartiles; whiskers, 1.5 times the interquartile range. Kruskal–Wallis test was performed to calculate the *P* value across different groups. Pearson correlation was performed to calculate the *P* value of all spots within each slide. Black dots: *P* < 0.05; gray dots: *P* ≥ 0.05. Red dotted line represents the correlation value of 0. **e**, **f** Representative images of the lung sections of hACE2 mice before and after SARS-CoV-2 infection detected by immunofluorescent staining. TREM2 and CD68 at d0 and d14 (**e**); FBP1 and CD68 at d0 and d14 (**f**). TREM2^+^ or FBP1^+^ macrophages were indicated by arrows. Scale bars, 50 μm. **g** Boxplot showing the proportion of *SLAMF9*^+^*SPP1*^+^, *TREM2*^+^, *FBP1*^+^, *FBP1*^+^*MKI67*^+^, and *MARCO*^+^*CD36*^+^*TREM2*^*–*^*FBP1*^*–*^ macrophages in *CD68*^+^ macrophages in scRNA-seq data of human lung autopsy samples of control (*n* = 8) and COVID-19 patients (*n* = 5). Center line, median; box bounds, first and third quartiles; whiskers, 1.5 times the interquartile range. Data are represented as mean ± SEM. Two-sided Wilcoxon test.

Both scRNA-seq and Stereo-seq data revealed that SARS-CoV-2 RNAs were nearly absent from hamster lungs at d14. Since *Cd38*^+^ and *Slamf9*^+^ macrophages did not exhibit expression of cell death-related genes, a critical question emerges, i.e., what is the destination of *Cd38*^+^ and *Slamf9*^+^ macrophages after viral clearance? Tight linkage between *Slamf9*^+^ macrophages and *F13a1*^+^, *Trem2*^+^, and *Fbp1*^+^ macrophages was revealed by CellRank analysis (Fig. 5a), indicating that *Slamf9*^+^ macrophages may differentiate into these macrophage subpopulations during the inflammation resolution stage.

*Trem2*^+^ and *Fbp1*^+^ macrophages were specifically enriched at d14 but were absent under normal physiological conditions in healthy controls, and were termed as AM-Trem2 (cluster 14, *Trem2*^hi^ *Spp1*^hi^), AM-Fbp1 and AM-Fbp1-Mki67 (cluster 15 and 20, *Fbp1*^hi^; Fig. 5b). Different from *Trem2*^+^ and *Fbp1*^+^ macrophages, *F13a1*^+^ macrophages (IM-F13a1, cluster 21) were enriched at d0 and d2 but depleted at d5 and d7 and partially restored at d14 (Supplementary Fig. S1a). Expression of *Trem2* (triggering receptor expressed on myeloid cells 2, related to fibrosis) and *Fbp1* (fructose-bisphosphatase 1, a key enzyme in gluconeogenesis) may modulate the metabolic state and the function of macrophages for inflammation resolution and tissue repair^38,39^. RNA velocity analysis suggested sequential state transitions from AM-Trem2 towards AM-Fbp1 and tissue-resident AM-Cd36 (the most frequent subtype of AMs at d0; Fig. 5c). We further conducted the trajectory and pseudo-time analysis based on Monocle2. The results were consistent with RNA velocity analysis and further indicated genes undergoing prominent temporal changes during the differentiation, including the down-regulation of fibrosis-related and inflammatory genes (i.e., *Trem2*, *Spp1*, *C1qa*, *Fcgr2b*, *Tnf*, etc), and the restoration of homeostasis-related genes (i.e., *Marco*, *Cd36*, etc; Supplementary Fig. S6a, b), consistent with the process of inflammation resolution and tissue repair.

Evidenced by the Stereo-seq data, AMs were the top cell type spatially co-localized with the new subpopulation of *Fbp1*^+^ AMs (Fig. 5d), significantly higher than other cell types, suggesting the potential role of *Fbp1*^+^ AMs in the replenishment of AMs. The proliferating *Fbp1*^+^ AMs (AM-Fbp1-Mki67, cluster 20; Fig. 5b) at d14 may again confirm the roles of *Fbp1*^+^ AMs in the replenishment of AMs and suggest the importance of macrophage proliferation in inflammation resolution and tissue repair in addition to the immune responses against viral infection^31^.

We validated the presence of these *Fbp1*^+^ AMs and *Trem2*^+^ AMs specifically emerging at the inflammation resolution stage (d14) by in situ RNA hybridization (Supplementary Fig. S6c), which was further supported by immunofluorescent staining in the lung sections of hACE2-mice infected by SARS-CoV-2 (Fig. 5e, f). We also validated the presence of these macrophages based on the publicly available human autopsy scRNA-seq dataset^40^. By employing a set of signature genes specifically expressed in *Slamf9*^+^, *Trem2*^+^, *Fbp1*^+^, and *Marco*^+^*Cd36*^+^ macrophages identified in our study (Supplementary Fig. S6d), we confirmed the enrichment of *SLAMF9*^+^, *TREM2*^+^, *FBP1*^+^, and *MARCO*^+^*CD36*^+^ macrophages and the proliferation of *FBP1*^+^ macrophages in the lung autopsy samples of COVID-19 patients (Fig. 5g). In contrast, autopsy samples from pulmonary bulla or lung cancers (stated as Control group) that were pathologically confirmed to be inflammation-free demonstrated only *FBP1*^+^ macrophages (Fig. 5g).

## Discussion

In this study, a high-resolution spatiotemporal landscape of SARS-CoV-2 pulmonary infection and inflammation resolution in hamsters was constructed, and the massive data revealed a new subpopulation of macrophages, i.e., *Slamf9*^+^ macrophages, which is at the core of SARS-CoV-2 clearance and thereafter inflammation resolution and immune homeostasis. The dynamics and mechanisms of *Slamf9*^+^ macrophages in clearing SARS-CoV-2 and resolving inflammation may be summarized as follows: (1) derived from peripheral monocytes with Mac-Cd38 as intermediates; (2) induced specifically by SARS-CoV-2 engulfment or infection because of the extremely high rates of SARS-CoV-2 RNA positivity in these cells; (3) proliferate actively to win the fight against SARS-CoV-2 intra-host transmission; (4) cell death-resistant, different from resident alveolar macrophages; (5) secrete chemokines to recruit neutrophils and clear SARS-CoV-2 together; (6) secrete IL-10 to induce an immunosuppressive niche that is co-localized by *Isg12*^+^*Cst7*^+^ neutrophils to prevent inflammation-induced damages; (7) differentiate into *Trem2*^+^ and *Fbp1*^+^ macrophages to further resolve inflammation; and (8) replenish IMs directly or AMs indirectly.

SLAM9 was first identified as CD84-H1 by our group from human DCs by automatic large-scale screening, which is widely expressed in immune cells, including DCs, monocytes, B cells, and T cells^41^. Our current study further extends the potential function of SLAMF9^+^ macrophages^42,43^, and also the SLAM-family receptors in viral infection and pulmonary diseases. Since single-cell spatiotemporal studies are inherently unbiased and observational, the hypotheses generated are more reliable than those based on limited indices and are more deserving of validation and further exploration through experimental studies. Our study revealed that *Slamf9*^+^ macrophages were specifically induced by SARS-CoV-2 infection. However, the conditions sufficient or necessary for the induction of *Slamf9*^+^ macrophages are still unknown, and further studies are needed to illustrate the metabolic, epigenomic, physical factors necessary for the induction and function of *Slamf9*^+^ macrophages. Similarly, proliferation of different macrophage subsets and neutrophils has been observed to be an important mechanism to act against SARS-CoV-2 infection and maintain immune homeostasis. However, the factors and conditions related to the macrophage and neutrophil proliferation in vivo are still unknown and require future experiments to illustrate. Previous studies have provided insights that SARS-CoV-2 could infect and replicate in human lung-resident macrophages evidenced by transcriptomic profiling and mechanistic studies^29,44^. *Cd274*^+^ and *Cst7*^+^ neutrophils were previously observed in the blood sample of COVID-19 patients and associated with disease severity^9,45^, confirming the clinical relevance of our finding and indicating the importance of this group of neutrophils in understanding COVID-19. But the detailed mechanisms of this group of neutrophils in SARS-CoV-2 clearance still need further experiments to substantiate. With long COVID-19 becoming an increasing global concern^46^, our identification of *Trem2*^+^ and *Fbp1*^+^ macrophages and their roles in inflammation resolution, immune homeostasis, and tissue repair is helpful for developing treatment for post-acute infection sequelae of COVID-19. However, the underlying factors critical for the functions of these macrophage subgroups still require clarification.

In summary, our study depicted the spatiotemporal dynamics of pulmonary immune homeostasis with SARS-CoV-2 infection in hamsters as a model at single-cell resolution and characterized the cellular and molecular events covering the whole process from viral infection, tissue damage, immune infiltration, viral clearance, to inflammation resolution and tissue repair. The identification of SARS-CoV-2 infection of *Slamf9*^+^ macrophage subpopulation provided actionable hypotheses for precisely understanding the mechanisms underlying lung injury, viral clearance, inflammation resolution, and immune homeostasis, which is insightful for developing anti-viral therapies. The rich information of our scRNA-seq and Stereo-seq data provides a comprehensive and insightful resource for understanding pulmonary immune homeostasis and developing anti-viral therapies in future.

## Materials and methods

### Ethics Statement

At the Institute of Laboratory Animal Science (ILAS) of Chinese Academy of Medical Sciences, the animal biosafety level 3 (ABSL-3) facility was used to accomplish all the experiments with Syrian hamsters (male and female, aged 8–10 weeks) and hACE2 mice (male and female, aged 6–11 months). All experiments were implemented according to the Animal Welfare Act and other regulations associated with animals and experiments. The Institutional Animal Care and Use Committee (IACUC) of the ILAS, Peking Union Medical College & Chinese Academy of Medical Sciences, evaluated and gave permission to all the protocols in these studies, including animal experiments (Approval number QC21003).

### Viruses

The SARS-CoV-2 virus (accession number is MT093631.2, SARS CoV-2/WH-09/human/2020/CHN) was propagated in Vero E6 cells and incubated in Dulbecco’s modified Eagle’s medium (Invitrogen, Carlsbad, United States) supplemented with 10% fetal bovine serum (Gibco, Grand Island, United States) at 37 °C with 5% carbon dioxide. Influenza virus (IAV) strain A/Puerto Rico/8/1981 H1N1 (PR8) were used as previously described^47^.

### Experimental hamsters

Specific-pathogen-free, 8–10-week-old male and female Syrian hamsters were intranasally inoculated with SARS-CoV-2 stock virus at 10^5^ 50% tissue culture infectious dose (TCID50) per mL (0.1 mL per animal)^23^. Hamsters intranasally inoculated with an equal volume of PBS were used as the healthy control (Ctrl) group. Hamsters were continuously monitored to record clinical symptoms, body weight, responses to external stimuli, and death. No animals were needed to be sacrificed to avoid undue suffering. Lung tissues of the infected group were collected at 2, 5, 7, and 14 days after infection (*n* = 5 per timepoint), and those of the Ctrl group were collected at 2 days (*n* = 5).

### Experimental mice

Specific-pathogen-free, 6–8-week-old female C57BL/6J mice were obtained from Beijing Vital River Laboratory Animal Technology Co., Ltd. (Beijing, China). Specific-pathogen-free, 6–11-month-old male and female hACE2 mice were obtained from the Institute of Laboratory Animal Sciences, Chinese Academy of Medical Sciences as described in our previous study^12^. For SARS-CoV-2 infection, the hACE2 mice were intranasally inoculated with SARS-CoV-2 stock virus at a dosage of 10^5^ TCID50, and those intranasally inoculated with an equal volume of PBS were used as healthy control (d0). The infected hACE2 mice were continuously observed to record body weight, clinical symptoms, responses to external stimuli, and death. For IAV infection, C57BL/6J mice were intranasally infected with 3 × 10^7^ plaque forming unit (PFU). Mice were dissected after certain days to collect lung tissues for further experiments.

### In situ RNA hybridization

In situ RNA hybridization was performed using the Advanced Cell Diagnostics RNAscope Multiplex Fluorescent Detection kit v2 (323100, Bio-Techne) according to the manufacturer’s instructions. Staining of hamster lung specimens was performed using paraffin-embedded sections (3–4 µm in thickness). For multiplex staining, the following probes were used: Mau-Fbp1 1153521-C3, Mau-Il10 1153551-C3, Mau-Mki67 1153561-C2, Mau-Trem2 1153571-C2, and Mau-Cd68 899591-C1. Slides were counterstained with Mounting Medium With DAPI-Aqueous (ab104139, Abcam). Mounted slides were imaged on a Leica DMi8 fluorescent microscope (Leica Biosystems).

### Multiplex immunofluorescence staining

All collected lung tissues from hACE2 mice were fixed in 10% formalin buffer solution (HT501128, Sigma-Aldrich), and paraffin-embedded sections (3–4 µm in thickness) were prepared according to routine operating procedure^48,49^. The immunofluorescence staining was performed using a PANO IHC kit (10004100100, Panovue, Beijing, China) following the manufacturer’s instructions^50^. Different primary antibodies were sequentially applied to examine specific cell markers, including anti-CD68 Polyclonal antibody (28058-1-AP, 1:800; Proteintech) and TREM2 Polyclonal antibody (13483-1-AP, 1:200; Proteintech), or FBP1 Polyclonal antibody (12842-1-AP, 1:100, Proteintech), or Osteopontin Polyclonal antibody (22952-1-AP, 1:200; Proteintech) and SLAMF9 polyclonal antibody (LM-2205R, 1:200; LMAI BIO), followed by HRP-conjugated secondary antibody incubation and tyramide signal amplification (TSA). The slides were microwave-treated after each cycle of TSA. Nuclei were stained with 4’-6’-diamidino-2-phenylindole (DAPI; Sigma-Aldrich) after antigen labeling. Stained slides were scanned using the 3D-histech (PANNORAMIC, 3DHISTECH, Hungary), which captures fluorescent spectra with identical exposure times using DAPI, FITC, TRICT and Cy5 channels, and the scans were combined to build a single stacked image.

### Sorting for lung macrophages

Lung tissues were first rinsed with PBS, minced into small pieces by mechanical dissociation, and incubated at 37 °C for 30 min in the digestion buffer, containing 2 mg/mL Collagenase I (17018029, Gibco), 2 mg/mL Collagenase IV (17104019, Gibco), 0.2 mg/mL DNase I (DN25, Sigma-Aldrich) and 10% FBS in RPMI 1640 medium. After this, the tissue digestion was blocked by adding RPMI 1640 medium containing 20% FBS and 1 mM EDTA, followed by filtration through a 40-µm cell strainer and centrifugation for 5 min at 500× *g* at 4 °C. Red blood cells were lysed in Red blood cell lysis buffer (R1010, Solabio) for 5 min, and washed with PBS buffer, followed by filtration through a 40-µm cell strainer and centrifugation for 5 min at 500× *g* at 4 °C. 5 × 10^5^ cells were isolated for each sample, which were Fc-blocked with CD16/32 antibody (101302, Biolegend). Cells were stained with Zombie viability dye (423113, Biolegend) for 10 min at room temperature, and then were stained with surface marker CD45-APC/Fire 750, CD64-PE/Cy7, MerTK-FITC, CD11b-APC, Ly6G-BV421, and SLAMF9-BV605 for 30 min at 4 °C. Then, the stained cells were washed twice for acquisition and sorting on a BD LSR Fortessa and SONY MA900. Data were analyzed by Flowjo.

### Quantitative RT-PCR

Total RNA was isolated from macrophages using RNAfast2000 kit (220011, Fastagen Biotech) according to the manufacturer’s instructions. ReverTra Ace qPCR RT Master Mix (FSQ-201, TOYOBO) was used to synthesize cDNA. SYBR Green Realtime PCR Master Mix (QPK-201, TOYOBO) was used for real-time PCR analysis on QuantStudio 3 (Applied Biosystems). The relative mRNA expression levels of *Slamf9, Ifnb1,* and virus were normalized to the expression of mouse *Actb* according to the 2^-ΔΔCt^ calculation method, and data were shown as mean ± SEM. The primer sequences were listed in Supplementary Table S1.

### scRNA-seq library preparation and sequencing

Single-cell suspension preparation, library construction and sequencing, raw data processing, and unsupervised cell clustering and annotations were performed according to the previous procedures^25^.

#### Single-cell suspension preparation

In the ABSL-3 laboratory, fresh lung tissues were first rinsed with PBS, minced into small pieces by mechanical dissociation, and incubated at 37 °C for 30 min in 10 mL of digestion buffer, containing 2 mg/mL Collagenase I (17018029, Gibco), 2 mg/mL Collagenase IV (17104019, Gibco), 0.2 mg/mL DNase I (DN25, Sigma-Aldrich) and 10% FBS in RPMI 1640 medium. After this, the tissue digestion was blocked by adding 3 mL of RPMI 1640 medium containing 20% FBS and 1 mM EDTA, followed by filtration through a 40-µm cell strainer and centrifugation for 5 min at 500 × *g* at 4 °C. Red blood cells were lysed in Red blood cell lysis buffer (R1010, Solabio) for 5 min, and washed with PBS buffer, followed by filtration through a 40-µm cell strainer and centrifugation for 5 min at 500 × *g* at 4 °C. Pellets were resuspended in cell resuspension buffer at a concentration of 1000 cells/μL for library preparation.

#### Library construction and sequencing

DNBelab C Series Single-Cell Library Prep Set (1000021082, MGI) was used as described previously^25^. In brief, high-quality single-cell suspension was used for droplet generation, cell lysis, and mRNA capture by microbeads were performed in the droplets, then emulsion breakage, beads collection, reverse transcription, finally the cDNA and droplet index were amplification to generate cDNA library and droplet index library respectively. All the processes were performed in the ABSL-3 laboratory till the cDNA products were gained. Library concentration was measured by use of Qubit™ ssDNA Assay Kit (Q10212, Thermo Fisher Scientific) and sequenced by DNBSEQ T10 sequencer in the China National GeneBank (Shenzhen, China).

### scRNA-seq data processing

#### Raw data processing

For DNBelab C4 data, PISA (v0.7, https://github.com/shiquan/PISA) was used for sequencing read filtering, and demultiplexing. The reference genome FASTA file and gene annotation file (GTF) for the Mesocricetus auratus genome (BCM_Maur_2.0, NCBI RefSeq assembly: GCF_017639785.1) and the SARS-CoV-2 genome (NCBI Reference Sequence: NC_045512.2) were downloaded from NCBI. These files were then integrated together as an inhouse hamster reference genome. The alignment tool STAR (v2.7.9a)^51^ was applied to map reads with the reference genome.

#### scRNA-seq data

The number of detected genes, the total UMI counts and proportion of mitochondrial gene counts per cell were used for quality control. Low-quality cells with <1000 UMI counts, <300 detected genes, or >5% mitochondrial gene counts were filtered. Cells with >60,000 UMI counts or >10,000 detected genes were filtered out to remove potential doublets and multiplets. Then scDblFinder (v1.9.3) was applied to further identify potential doublets with its random methods (https://github.com/plger/scDblFinder).

#### Unsupervised cell clustering and annotations

Clustering analysis of the scRNA-seq dataset was performed using the Seurat package (v4.0.5)^52^ and the R program (v4.1.0). UMI count matrices of different samples were merged together for analysis, and then normalized (LogNormalization function, scale.factor = 10000) and scaled with Seurat. 2000 highly variable features were identified and a PCA matrix with 50 components was constructed. For clustering cells precisely, we applied two round clustering to the dataset. The first-round clustering (resolution = 0.4) identified ~5 major cell types including non-immune cells, myeloid cells, T cells, B cells and neutrophils. Clustering results were displayed by UMAP^53^ dimension reduction analysis. A second round of clustering was applied to each major cell type based on a procedure similar to the first round of clustering. For the calculation of cluster markers, we used the sc.tl.rank_genes_groups function of scanpy, and retained the remaining genes for cluster identification after setting certain parameters (threshold > 0.25 and *P*val_adj < 0.05). Annotation of the final clusters was based on the known cell markers. Finally, 142,965 identified cells were remaining for downstream analysis. 79 clusters were finally obtained with manual curation, representing different cell subpopulation. Additionally, we found that 17,149 cells in all cells were detected with viral reads (UMI > 0).

#### Temporal distribution of different cell subpopulations

The temporal distribution of cell populations was indicated by Ro/e value, which was calculated according to the previous procedures^25^ by the following formula previously described (https://github.com/Japrin/STARTRAC)^54^:

$${Ro}/e=\frac{{observed}}{{expected}}$$

In which the observed variable is the cell number of a given ${{subpopulation}}_{i}$ at a specific ${{timepoint}}_{j}$, and the expected variable is calculated by the following formula, which represents the expected cell number distribution for ${{subpopulation}}_{i}$ at ${{timepoint}}_{j}$:

$${expected}=\frac{{N}_{i}}{{N}_{{all}}}\times {M}_{j}$$

In which ${N}_{{all}}$ is the total cell number of all subpopulations, ${N}_{i}$ is the cell number of the given ${{subpopulation}}_{i}$, and ${M}_{j}$ is the cell number of all subpopulations at the specific ${{timepoint}}_{j}$. *Ro/e* > 1, it suggests that cells of the specific subpopulation are more frequently observed than random expectations at the specific timepoint (enrichment). If *Ro/e* < 1, it suggests that cells of the specific subpopulation are observed with less frequency than random expectations at the specific timepoint (depletion).

#### Ligand–receptor analysis by CellChat

Cell types of interest (spatially co-localized) were selected for CellChat analysis. To investigate intercellular interactions among multiple cell types, the CellChat package (v2.1.2) was used to predict active ligand–receptor interactions^55^. We performed ligand–receptor interactions analysis among Mac-Slamf9, Mac-Slamf9-Mki67, and neutrophil subpopulations. We obtained expression lognormalized data matrix as input, and then analyzed with default parameters in https://github.com/sqjin/CellChat. Circle plots were draw with netVisual_circle function. The role of signaling between different cell subpopulations were shown with netAnalysis_signalingRole_heatmap function. The input murine reference ligand–receptor pair list was obtained by CellChatDB.mouse function.

#### Differential gene expression and Gene Ontology enrichment analysis

To investigate the effects of viral RNA in one subpopulation, we identified differentially expressed genes between viral RNA positive and negative cells by performing two-sided unpaired Wilcoxon tests based on the tl.rank_genes_groups function of the python package Scanpy (v1.8.1) (method = “wilcoxon”, corr_method = “benjamini-hochberg”)^56^. Differentially expressed genes for subsequent enrichment analysis were further identified by two rules, i.e., log_2_foldchanges > 0.5 and *Padj* < 0.05. Over-representation analysis was processed in different subpopulations using enrichGO (OrgDb = org.Mm.eg.db, pAdjustMethod = “BH”) function of the Cluster Profiler R package (v4.0.5)^57^.

#### RNA velocity analysis

The rates of gene splicing and degradation can be estimated by leveraging the relative abundance of nascent (unspliced) and mature (spliced) mRNA. The raw count matrices for unspliced and spliced mRNA were computed using velocyto (v0.17.16) (http://velocyto.org/velocyto.py/index.html) with the gtf annotation and bam file as inputs. To explore the intercellular relationships within main lineages, RNA velocity analysis was performed on alveolar macrophages or neutrophils using the scVelo package (v0.2.4)^58^. The resulting data was further analyzed using scanpy (v1.7.2)^56,59^. First, it involved the selection of 2000 highly variable features through the utilization of the scanpy.pp.highly_variable_genes() function. Second, the moments were computed in PCA space using the scvelo.pp.moments() function with a parameter value of n_neighbors = 30, n_pcs = 30. The RNA velocity was calculated using the scvelo.tl.velocity() function with mode = “stochastic” and then a velocity graph was constructed employing the scvelo.tl.velocity_graph() function with default parameters. Finally, the velocities were projected using scv.pl.velocity_embedding_stream() with basis = “umap”.

#### Gene set score analysis

To evaluate neutrophil maturation and function scores, we calculated the expression level of individual neutrophils with regarding predefined genesets^60^. AddModuleScore function with default parameters was applied in this process.

#### Pseudo-time analysis of macrophages

To analyze dynamic biological processes among AM-Trem2, AM-Fbp1, AM-Fbp1-Mki67, and AM-Cd36, we used the monocle R package (v2.20.0)^61–63^. We extracted UMI counts matrix of four subpopulations motioned above as input. We processed the pseudo-time analysis followed tutorial described in http://cole-trapnell-lab.github.io/monocle-release/docs/ with default parameters. Additionally, we sorted 517 genes out with parameters of mean_expression ≥0.1 and dispersion_empirical ≥1 * dispersion_fit for constructing single cell trajectories.

#### Monocyte/macrophage cell fate inference analysis

We used CellRank (v2.0.2) to conduct cell fate inference analysis on single-cell transcriptomic data obtained from monocyte-macrophage populations spanning from d7 to d14^64^. Our analysis employed the RealTimeKernel method of CellRank, which enables the prediction of single-cell differentiation trajectories and transition probabilities using a Markov chain model that incorporates the actual experimental time. By leveraging the resulting single-cell transition probability matrix, we investigated the process of fate determination at the population level for monocyte-macrophage populations during the specific timeframe of d7 to d14.

#### Cell subpopulation similarity analysis between Syrian hamsters and COVID-19 patients

We also analyzed snRNA-seq data derived from autopsy lung tissues of COVID-19 patients^40^, to confirm whether subpopulation changes were similar between humans and Syrian hamsters. With homologene R package (v 1.4.68.19.3.27), we selected whole 13,419 homologous genes, including *MARCO*/*Marco*, *CD68*/*Cd68*, *CD36*/*Cd36*, *TREM2*/*Trem2*, *FBP1*/*Fbp1*, *SLAMF9*/*Slamf9* and *MKI67*/*Mki67*. We applied a set of signature gene combinations to represent the cell types (Mac-Slamf9: *CD68*^+^*SLAMF9*^+^*SPP1*^+^; AM-Trem2: *CD68*^+^*TREM2*^+^; AM-Fbp1: *CD68*^+^*FBP1*^+^; AM-Fbp1-Mki67: *CD68*^+^*FBP1*^+^*MKI67*^+^; AM-Cd36: *CD68*^+^*MARCO*^+^*CD36*^+^*TREM2*^*–*^*FBP1*^*–*^), which performed well in the scRNA-seq data generated by our study. Then we detected cell proportions with signature gene combinations expression.

### Stereo-seq library preparation and sequencing

Tissue processing, library construction and sequencing, Stereo-seq raw data processing, quality control, and batch effect correction of bin80 spatial Stereo-seq data, and spatial transcriptomics deconvolution by Redeconve were performed according to the previous procedures^25^.

#### Stereo-seq raw data processing

Fastq files were generated using a MGI DNBSEQ-T10 sequencer. *Mesocricetus auratus* genome (BCM_Maur_2.0) and SARS-CoV-2 genome were integrated as one reference for read mapping by STAR. One base mismatch was allowed to correct sequencing and PCR errors. Mapped reads were counted and annotated using a BGI-developed open-source pipeline SAW (https://github.com/BGIResearch/SAW).

#### Binning data of spatial Stereo-seq data

Because one stereo-chip contained millions of DNBs (diameter: 220 nm), we merged adjacent 80 × 80 DNBs to one bin80 spot (40 μm resolution) as a fundamental unit for downstream analysis, including unsupervised clustering, Redeconve deconvolution calculation, and spatial correlation calculation. Additionally, matrices of different spatial Stereo-seq samples were normalized to 1,000,000 counts before analysis.

#### Quality control and batch effect correction of bin80 spatial Stereo-seq data

Stereo-seq data were quality-controlled by filtering low-quality spots for each chip based on the number of detected genes and the total UMI counts in each spot. We got 22,927 bin80 spots per section, 557 genes, and 1115 UMI counts per spot of 15 histologically intact bin80 chips after quality control. Then bin80 Stereo-seq data matrices were merged, log-normalized, and scaled by the R package Seurat, just like scRNA-seq matrices. We identified 2000 highly variable features across all merged spots and calculated a PCA matrix with 20 components. RunHarmony function in the Harmony package was used to do batch effect correction for different chips^65^.

#### Spatial transcriptomics deconvolution by Redeconve

We conducted deconvolution analysis of the spatial transcriptomics slides at the bin80 level with the deconvoluting function in the R package Redeconve (v. 0.0.0.9008)^66^, with the average expression profiles of 79 subpopulations identified by our scRNA-seq data as reference. The raw signal from Stereo-seq was first divided into 40 μm × 40 μm grids (i.e., bin80). Genes encoding hemoglobins and rRNAs were excluded from deconvolution analysis because scRNA-seq data excluded red blood cells. The abundance of 79 cell subpopulations within each grid were calculated by using Redeconve with the hyperparameter setting to ten times the suggested value by Redeconve.

#### Spatial correlation calculation between cell subpopulations

After obtaining the cell abundance matrix of each Stereo-seq slide, Pearson’s correlation was used to calculate the spatial correlation between each pair of cell subtypes (calculated through the coloc.corr function in the R package Redeconve). We merged the 79 cell subtypes into 14 major cell types and then calculated Pearson’s correlation coefficients between genes (or cells) and the 14 major cell types with the cor.test function in R package.

### Statistical analysis

Statistics were processed with R statistical software v4.1.2 unless otherwise specified. Unpaired two-sided Student’s *t*-test, two-sided Wilcoxon test, and Kruskal–Wallis test were used as indicated.

## Supplementary information

Supplementary Figures

## Data Availability

The data that support the findings of this study have been deposited into CNGB Sequence Archive (CNSA) of China National GeneBank DataBase (CNGBdb) with accession numbers CNP0002742 and CNP0002978. All other data are included in the article and/or Supplementary information.
