# Supplementary material for: Single-cell spatiotemporal analysis of the lungs reveals Slamf9+ macrophages involved in viral clearance and inflammation resolution
Source: Cell Discov. 2024 Oct 16;10:104. doi: 10.1038/s41421-024-00734-4 (PMC11484945; doi:10.1038/s41421-024-00734-4)
Supplement: Supplementary file 1 — Supplementary Figures [file 41421_2024_734_MOESM1_ESM.pdf]

## Supplementary Information

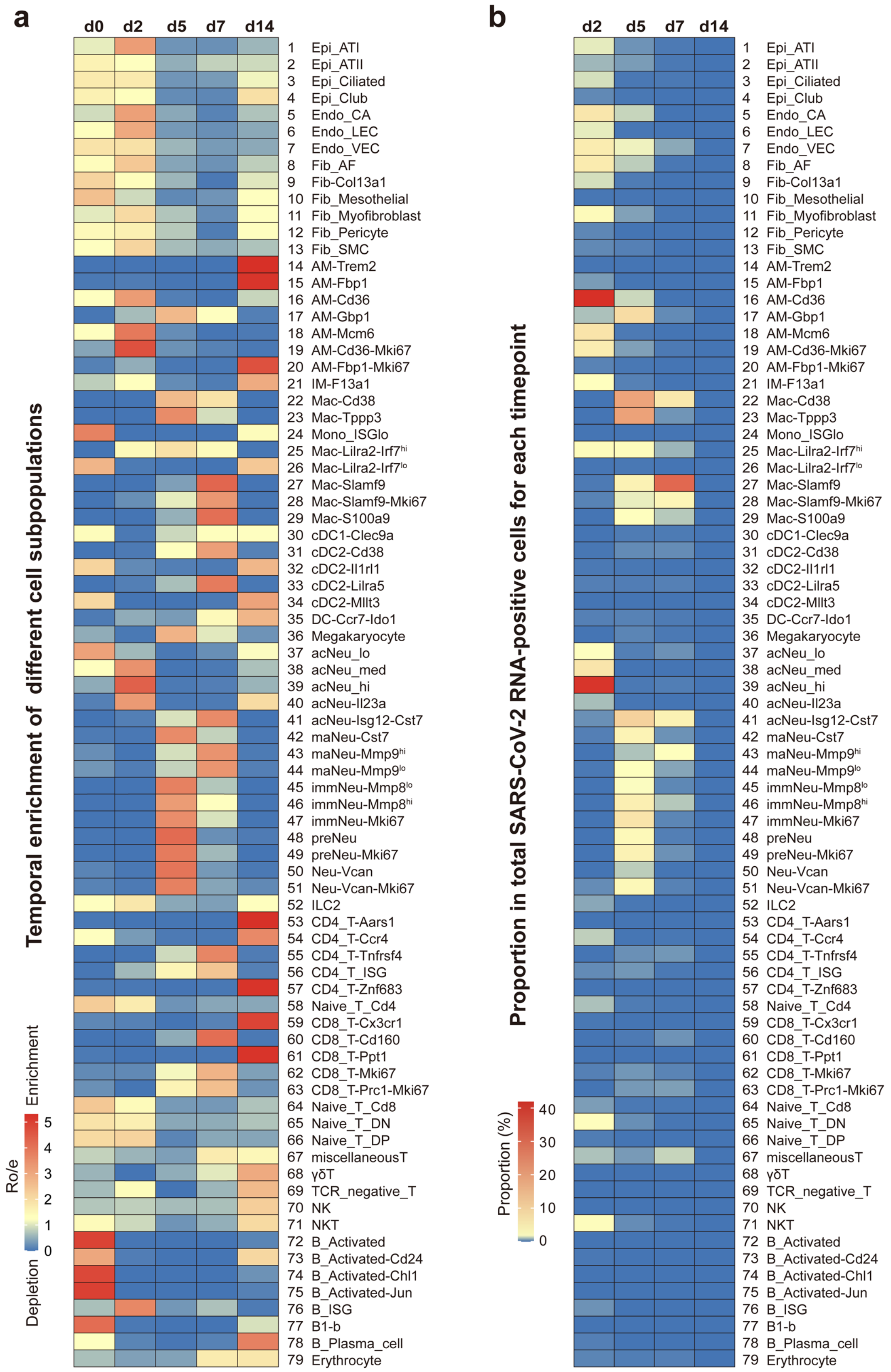


**Supplementary Fig. S1 Temporal signatures of 79 subpopulations before and after SARS-CoV-2 infection**

- **a.** Heatmap showing the temporal distribution of all 79 scRNA-seq cell subpopulations at d0, d2, d5, d7 and d14. Ro/e>1, enrichment; Ro/e<1, depletion.
- **b.** Heatmap showing the proportion of virus-positive cells of all 79 scRNA-seq cell subpopulations in total SARS-CoV-2 RNA-positive cells at d2, d5, d7 and d14.

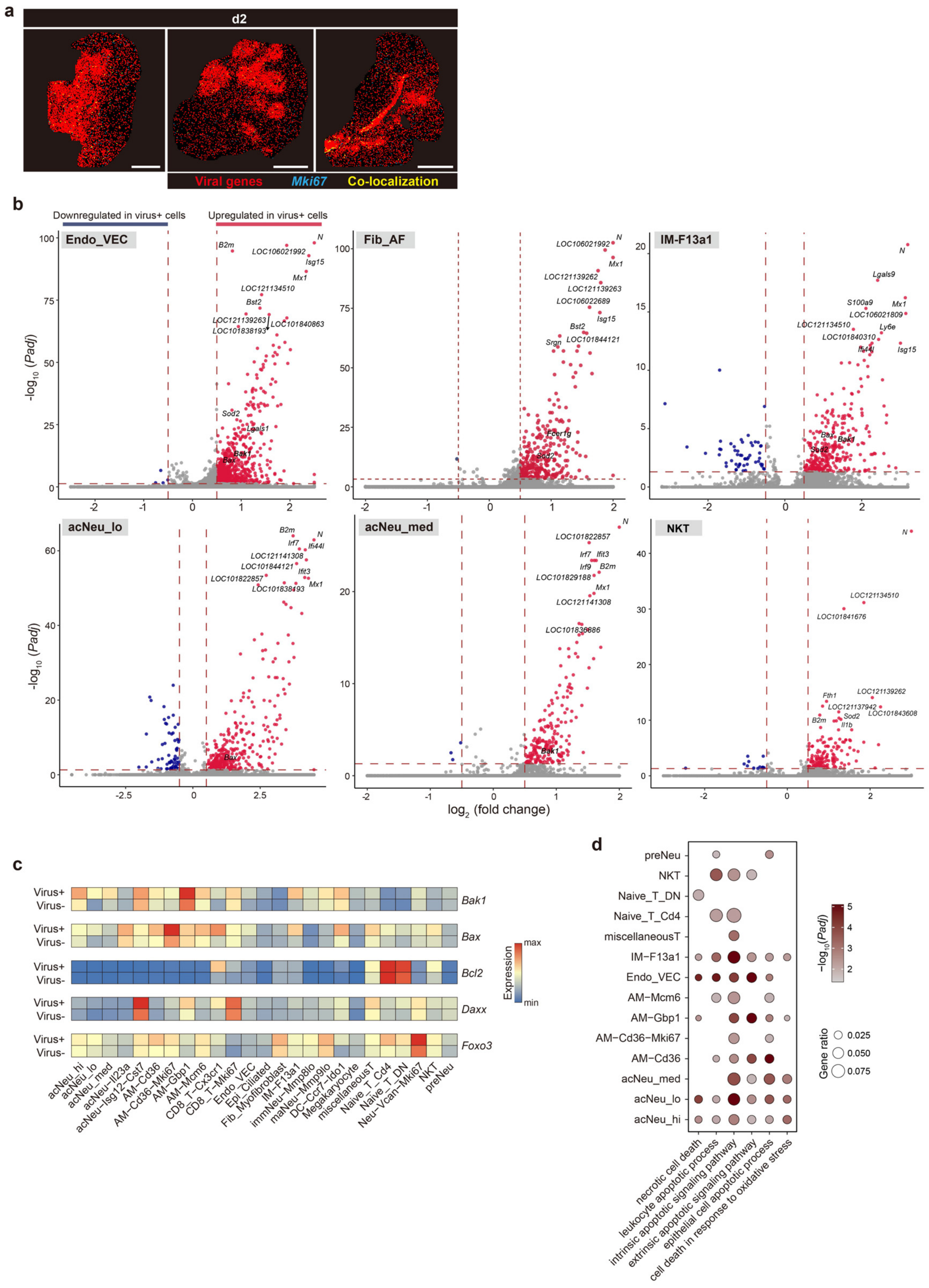


**Supplementary Fig. S2 Acute damages of multiple cell types caused by SARS-CoV-2 infection**

**a.** Spatial distribution of viral genes and *Mki67*, represented by three lung sections at d2. Scale bar, 2 mm.

**b.** Volcano plots of differentially expressed genes between virus-positive and virus-negative cells in representative cell subpopulations (cutoff: ($|Log_2FC| > 0.5$, *Padj* < 0.05).

**c.** The expression of cell death-related genes *Bak1*, *Bax*, *Bcl2*, *Daxx* and *Foxo3* in virus-positive and virus-negative cells of representative cell subpopulations.

**d.** Cell death-related GO terms enriched in upregulated genes of virus-positive cells of representative cell subpopulations.

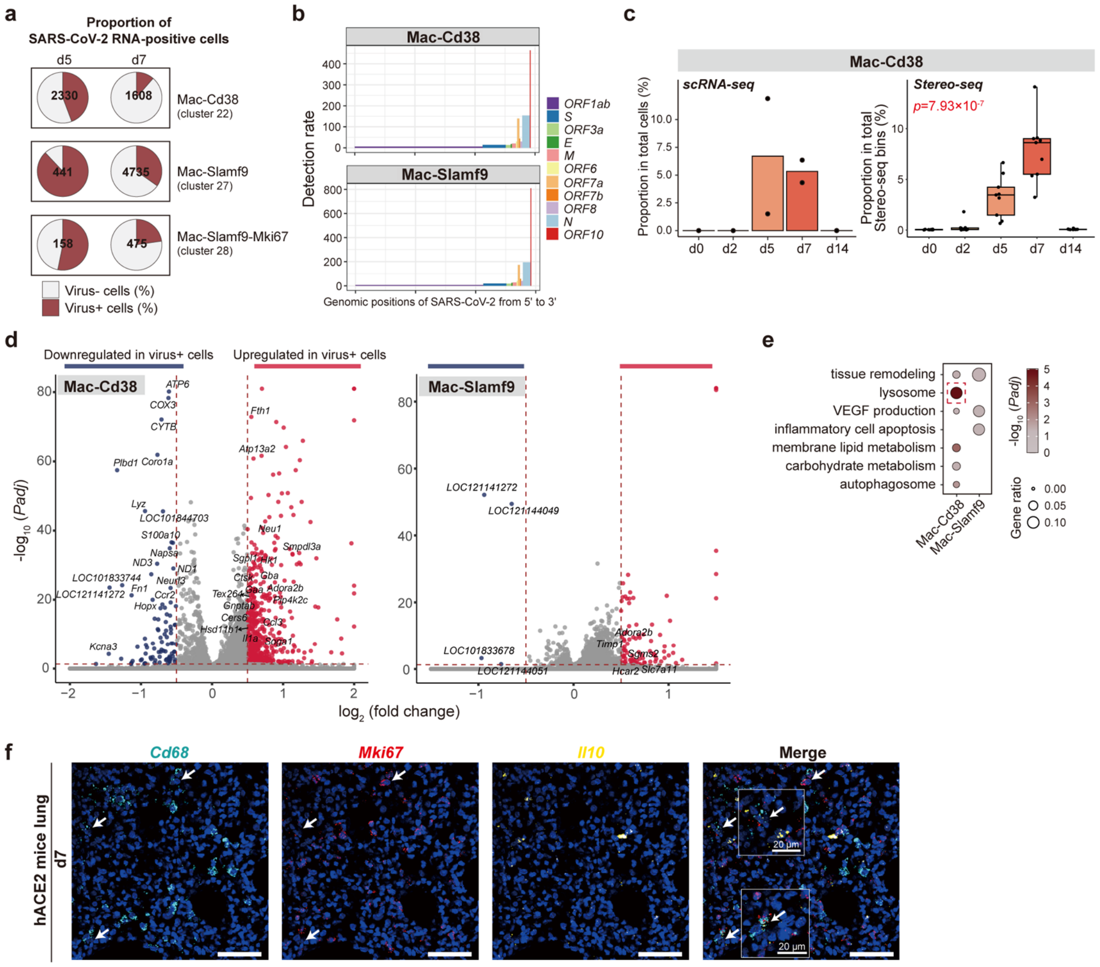


**Supplementary Fig. S3 Signatures of *Cd38*⁺ and *Slamf9*⁺ macrophages after infection**

**a.** Pie chart showing the relative proportion of virus-positive cells within *Cd38*⁺, *Slamf9*⁺ macrophages and proliferating *Slamf9*⁺ macrophages subpopulations at d5 and d7. Total cell number of each cluster at each timepoint is labeled. More than 80% of *Slamf9*⁺ macrophages were virus-positive at d5.

**b.** Detection rates of SARS-CoV-2 genes in *Cd38*⁺ and *Slamf9*⁺ macrophages. Given a viral gene $g_v$, the detection rate is defined as the ratio of the number of $g_v$-positive cells to the total cells of the specific cell type and then normalized by the gene length in the SARS-CoV-2 genome and multiplied by $10^6$.

**c.** Left, bar chart showing the proportion of *Cd38*⁺ macrophages in total lung cells detected in two replicated hamsters by scRNA-seq. Right, boxplot showing the proportion of *Cd38*⁺ macrophages in total Stereo-seq bin80-bins of nine lung slides at each timepoint. Data are represented as mean±SEM (n=9 slides per timepoint).

Center line, median; box bounds, first and third quartiles; whiskers, 1.5 times the interquartile range. Kruskal-Wallis test.

**d.** Volcano plot of differentially expressed genes between virus-positive and virus-negative cells of *Cd38*$^{+}$ and *Slamf9*$^{+}$ macrophages (cutoff: ($|Log_2FC| > 0.5$, *Padj* $< 0.05$).

**e.** GO terms enriched in upregulated genes in virus-positive AM-Cd36, *Cd38*$^{+}$, *Slamf9*$^{+}$ macrophages. GO terms related with subpopulation functions mentioned are indicated by dashed line.

**f.** Representative images of proliferating *Slamf9*$^{+}$ macrophages (by detecting *Cd68*, *Mki67* and *Il10* RNA) in the alveoli regions of hACE2 mice lung slides at d7 detected by RNAscope. Proliferating macrophages are indicated by arrows and magnified images. Scale bar, 50 μm.

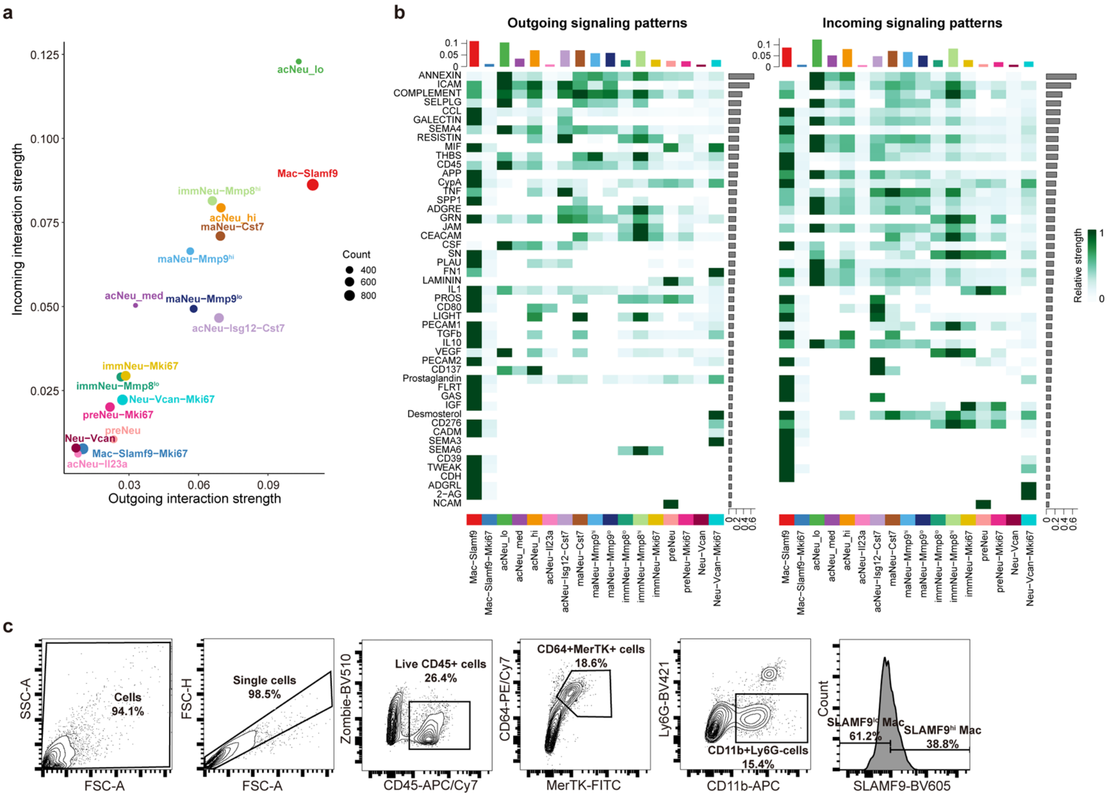

**Supplementary Fig. S4 Crosstalk among *Slamf9*$^{+}$ macrophages and neutrophil subpopulations**

**a.** Outgoing and incoming interaction strength of *Slamf9*$^{+}$ macrophages, proliferating *Slamf9*$^{+}$ macrophages, and 15 neutrophil subpopulations.

**b.** Heatmap displaying the outgoing and incoming interaction strength among these subpopulations, as determined through CellChat.

**c.** Gating strategies of flow cytometry sorting for SLAMF9$^{hi}$ and SLAMF9$^{lo}$ macrophages in the l lungs of C57BL/6J mice.

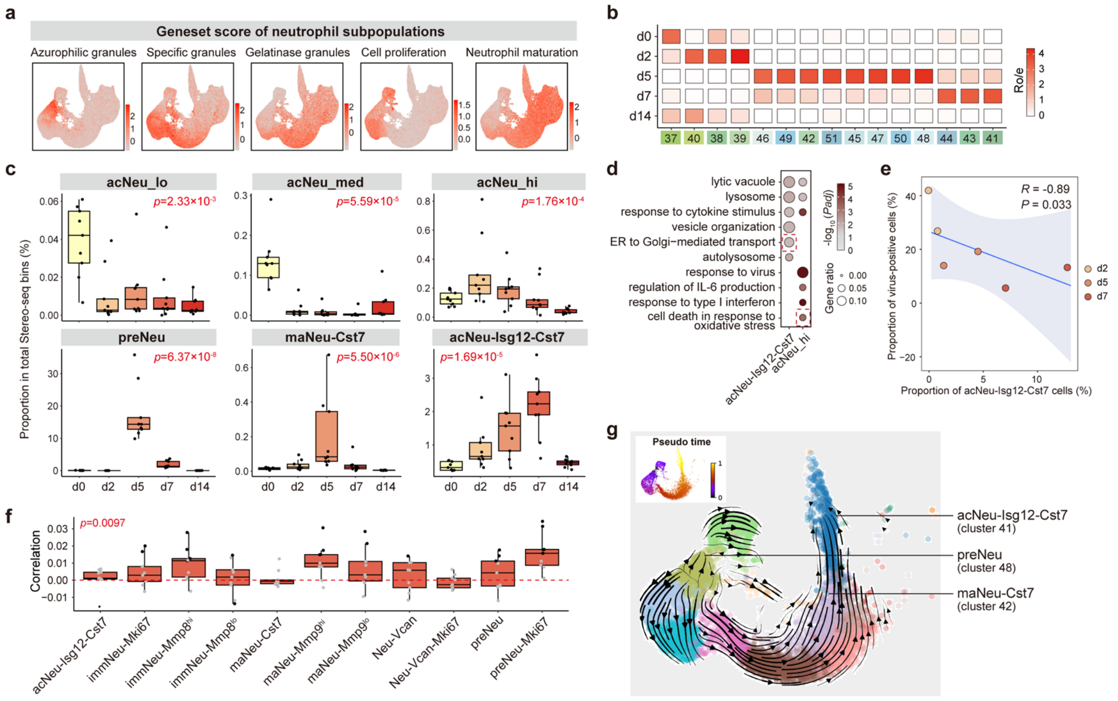


**Supplementary Fig. S5 Neutrophil dynamics during SARS-CoV-2 infection**

**a.** UMAP projection showing geneset scores of neutrophil subpopulations.

**b.** The temporal distribution of neutrophil subpopulations. Ro/e>1, enrichment; Ro/e<1, depletion.

**c.** Box plot showing the proportion of representative neutrophil subpopulations in total Stereo-seq bin80-bins of nine lung slides at each timepoint. Data are represented as mean±SEM (n=9 slides per timepoint). Center line, median; box bounds, first and third quartiles; whiskers, 1.5 times the interquartile range. Kruskal-Wallis test.

**d.** GO terms enriched in upregulated genes of virus-positive highly-activated neutrophils and *Isg12*$^{+}$*Cst7*$^{+}$ neutrophils. GO terms related with subpopulation functions mentioned are indicated by dashed line.

**e.** Pearson correlation between the proportion of virus-positive cells and *Isg12*$^{+}$*Cst7*$^{+}$ neutrophils in 12 hamsters (d2, d5 and d7).

**f.** Box plot showing the spatial correlation of different neutrophils subpopulations with viral genes. Data are represented as mean±SEM (n=9 slides per timepoint). Center line, median; box bounds, first and third quartiles; whiskers, 1.5 times the interquartile range. Kruskal-Wallis test was performed to calculate the p value across different groups. Pearson correlation was performed to calculate the p value

of all spots within each slide. Black dots: p value < 0.05; grey dots: p value ≥ 0.05. Red dotted line represents the correlation value of 0.

**g.** RNA velocity analysis of neutrophil subpopulations. Arrow direction indicates the potential directions of state transitions.

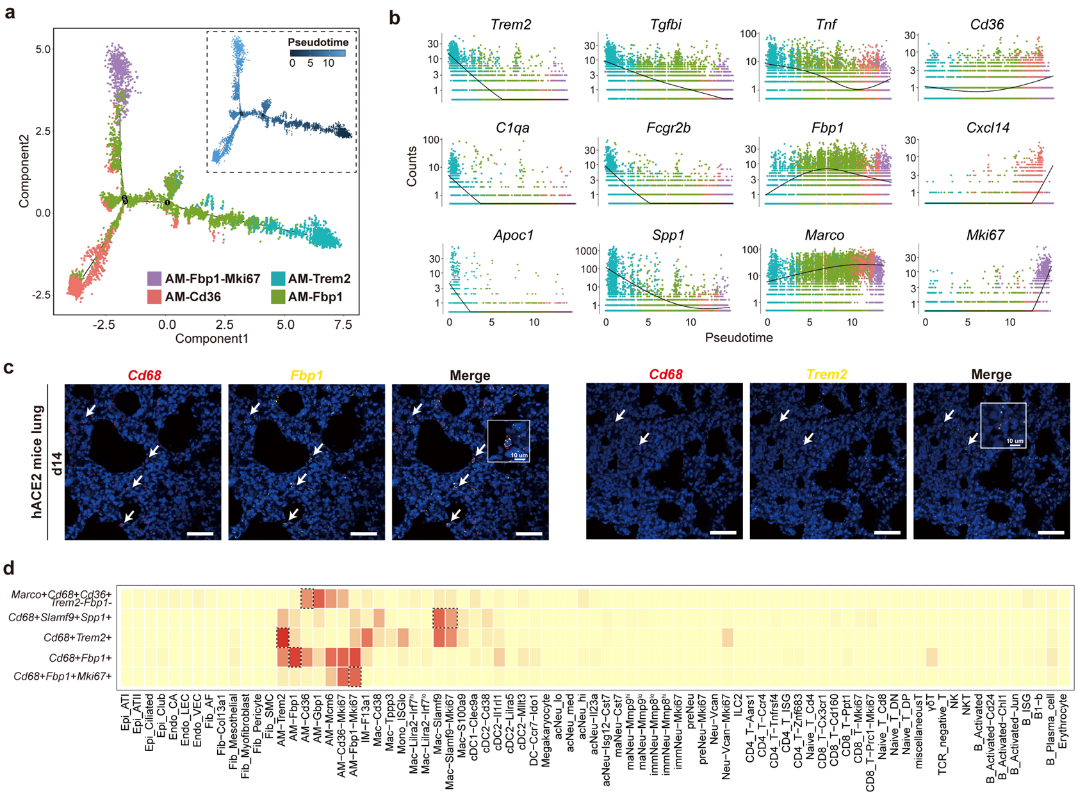


**Supplementary Fig. S6 Signatures of *Fbp1*$^{+}$ AMs and *Trem2*$^{+}$ AMs for inflammation resolution and tissue repair**

**a.** Pseudo-time trajectory initiated from AM-Trem2 towards AM-Fbp1, AM-Fbp1-Mki67 and AM-Cd36 predicted by Monocle2.

**b.** Expression of representative genes in the route along the pseudo-time.

**c.** Representative images of *Fpb1*$^{+}$ AMs and *Trem2*$^{+}$ AMs in the alveoli regions at d14 detected by RNAscope. *Fpb1*$^{+}$ AMs and *Trem2*$^{+}$ AMs are indicated by arrows and magnified images. Scale bar, 50 μm.

**d.** Heatmap showing the expression of signature genes of *Slamf9*$^{+}$, *Trem2*$^{+}$, *Fbp1*$^{+}$, *Fbp1*$^{+}$*Mki67*$^{+}$, and *Marco*$^{+}$*Cd36*$^{+}$ macrophages in all subpopulations identified in our study, indicated by dashed line.

**Supplementary Table S1. Primer sequences for qRT-PCR**

| Name | Sequence |
|---|---|
| *mIfnb1*-Fw | TCACCTACAGGGCGGACTTC |
| *mIfnb1*-Rev | TCTCTGCTCGGACCACCATC |
| *mSlamf9*-Fw | CAAAACAACATTGCCATCGTGA |
| *mSlamf9*-Rev | GCTAATATGCAGGGAGTAGCTG |
| *IAV-Fw* | GGCCGACTACACTCTCGATGA |
| *IAV-Rev* | TGTCTTATGGTGAATGACCTGGTTT |
| *mActb*-Fw | AGTGTGACGTTGACATCCGT |
| *mActb*-Rev | GCAGCTCAGTAACAGTCCGC |
